# Hypothalamic-pituitary-adrenal axis activity and its relationship to the autonomic nervous system in patients with psychogenic erectile dysfunction

**DOI:** 10.3389/fendo.2023.1103621

**Published:** 2023-03-03

**Authors:** Jinzhou Xu, Yinwei Chen, Longjie Gu, Xiaming Liu, Jun Yang, Mingchao Li, Ke Rao, Xiyuan Dong, Shulin Yang, Bo Huang, Lei Jin, Tao Wang, Jihong Liu, Shaogang Wang, Jian Bai

**Affiliations:** ^1^ Department of Urology, Tongji Hospital, Tongji Medical College, Huazhong University of Science and Technology, Wuhan, Hubei, China; ^2^ Reproductive Medicine Center, Tongji Hospital, Tongji Medical College, Huazhong University of Science and Technology, Wuhan, Hubei, China

**Keywords:** psychogenic erectile dysfunction, autonomic nervous system, heart rate variability, HPA axis, cortisol, perceived stress

## Abstract

**Background:**

Psychological stress and its two stress response systems, the hypothalamic-pituitary-adrenal (HPA) axis and the autonomic nervous system (ANS), are closely related to psychogenic erectile dysfunction (pED). However, the analyses of perceived stress and stress systems in pED patients need to be more in-depth, especially the interactions between them.

**Methods:**

Our study included 75 patients with pEDs and 75 healthy men. The International Index of Erectile Function-5 (IIEF-5) and the 10-item Perceived Stress Scale (PSS-10) were used for assessing the severity of ED and perceived stress. All participants collected saliva samples on three consecutive days at eight specific times with strict reference to the time of morning awakening for measuring cortisol parameters and wore electrocardiography for 24 h to derive heart rate variability (HRV).

**Results:**

The PSS-10 scores of pED patients were significantly higher than the control group (*p*<0.001). Although PSS-10 and IIEF-5 scores were negatively correlated in pED patients, there was no statistical significance between them (*r*=−0.049, *p*=0.677). Compared with the control group, the HRV parameters of pED patients were significantly increased in LF/HF ratio (*p*=0.014) but significantly decreased in LF, HF, and pNN50 (*p*<0.001). However, the two groups had no statistically significant differences in cortisol variables (all *p*>0.05). The interaction between sympathovagal modulation (HF, rMSSD) and cortisol awakening response (CAR AUCi) explained significantly greater variance in perceived stress than either stress system alone. Higher parasympathetic activity combined with a higher cortisol awakening response was associated with greater perceived stress.

**Conclusion:**

Our results suggested that the interrelation between ANS and HPA axis activity might enhance our comprehension of how stress affected the physical and mental health of pED patients.

## Introduction

Erectile dysfunction (ED) is the most common male sexual disorder, which refers to the persistent inability to attain or/and maintain an adequate penile erection to complete satisfactory sexual intercourse (1). Psychogenic ED (pED) is a primary subtype of ED caused by mental and psychosocial factors, which is diagnosed in the exclusion of organic factors and has severe impacts on the overall psychological health and quality of life (QOL) of patients and their partners (2). It even brings huge socio-economic burdens (3). ED is highly prevalent in modern societies, affecting approximately 30% of young men (4). pED patients account for 73.6% of ED patients under 40 years old in China (5). In recent years, pED has been the focus of researchers due to its extreme importance and high incidence (3).

Stress represents a necessary response that maintains *in vivo* homeostasis upon exposure to the threat of the environment and events (6). Chronic psychological stress has detrimental effects on both physical and mental health (7). It is known to cause physiological distress, leading to body balance perturbations associated with various metabolic and immune dysfunctions (8). Recent experimental evidence suggests that chronic stress is also closely related to ED (9–12). For instance, chronic stress might lead to the development of ED by reducing nitric oxide synthase expression in the penile constitution (9). The corpus cavernosum tissues of male rats under long-term stress had morphological changes (10). Chronic psychological stress impaired the neurogenic and endothelium-dependent relaxation function of the rabbit corpus cavernosum, resulting in ED (11). Additionally, men engaged in stress management had statistically significant reductions in perceived stress scores compared with men treated with tadalafil alone (12). Meanwhile, even if mental stress is not the cause of ED, ED can lead to psychological stress and further aggravate ED symptoms (13).

Psychological stress triggers a cascade of pathophysiological events mediated by the autonomic nervous system (ANS) and the hypothalamic-pituitary-adrenal (HPA) axis (14, 15). These two neural stress systems coordinate the responses of many other physiological systems to stressors, including the immune and cardiovascular systems, allowing the body to return to homeostasis (14). Dysregulation of the HPA axis or ANS can significantly disrupt homeostasis, causing cacostasis or allostasis, with various clinical manifestations. This could be a potential mechanism contributing to the pathogenesis of pEDs. However, so far, only a few studies have reported the changes in the ANS system in patients with ED. A previous study has shown that ED patients who were not classified by etiology exhibited relatively lower parasympathetic activity (16). Patients with pED further demonstrated significant cardiac sympathetic hyperactivity and severity-dependent vagal impairment (17). No reports have been found for the relationship between ED or pED and HPA axis change. Therefore, we intended to study the changes in these two stress response systems in pED patients simultaneously.

It is also worth noting that the ANS and HPA axis are highly coordinated and physically interconnected (18). The ANS and HPA axis activation in response to stress follows a coordinated, transient sequence. The ANS rapidly promotes physiological changes through the synaptic transmission of its two branches, the sympathetic and parasympathetic nervous systems. The parasympathetic nervous system promotes the sympathetic response to stress by eliminating its inhibitory effect, which enables physiological changes, including releasing norepinephrine by the locus coeruleus and stimulating sympathetic preganglionic neurons to increase heart rate (18). Whereas the HPA axis, as a hormonal system, exerts corresponding regulatory effects on the body a few minutes after activation. The HPA axis is initiated by releasing the corticotrophin-releasing hormone from the paraventricular nucleus of the hypothalamus, which leads to a cascade of endocrine events that ultimately lead to the release of cortisol from the adrenal cortex (18). Cortisol affects immune and metabolic systems and enhances the ANS activity, such as increasing the sympathetically mediated cardiovascular response to stress, manifested by an increased heart rate (19). The ANS works with the HPA axis to form biological and behavioral homeostasis. Thus, exploring the interrelation between ANS and HPA axis can provide more insight into the association between psychological stress and pED (18).

To deepen our understanding of the complexity of psycho-neuro-endocrine interactions in pED patients, in the present study, we aimed to determine whether and how both ANS and HPA axis changed in patients with pED and tested the hypothesis that inter-relation models, which included ANS and HPA axis interactions, would explain perceived stress better than models with ANS or HPA axis singularly.

## Materials and methods

### Participants

The study was carried out at the Urology Male Clinic of our hospital and ran from March 2016 to August 2022. Patients were interviewed to complete the Chinese version of the International Index of Erectile Function 5 (IIEF-5) (20). Inclusion criteria were partnered sexual activity, history of psychogenic ED for at least six months, a score of 5–21 in the IIEF-5 system, no conscious penile erection, poor hardness or non-lasting erection, and inability to complete normal sexual life; >18 or <50 years of age; genital examination showing no obvious developmental deformity; normal development of secondary sexual characteristics. Patients with vasculogenic and neurogenic ED (neurologic disease, pelvic surgery) were excluded. Participants using medications known to interfere with cardiovascular or endocrine function, such as tricyclic antidepressants and corticosteroids, were excluded. In addition, exclusion criteria also included other sexual dysfunction or sex hormone abnormalities or diseases that may influence ANS and HPA axis; psychosis; peripheral vascular disease; diabetes; spinal cord injury; coronary heart disease; hypertension; a history of alcohol or drug abuse, etc. All patients did not use drugs affecting sexual function in the past six months and were not administered drugs or other methods for ED treatment within three months. Healthy men were recruited as controls from advertisements in the communities surrounding the hospital.

### Perceived stress

Perceived stress was assessed using the Perceived Stress Scale, the most widely used psychometric instrument to measure the perception of stress. Participants were administered the 10-item Perceived Stress Scale (PSS-10) (21), an abbreviated version of the 14-item Perceived Stress Scale with high validity and reliability, assessed using a 5-point Likert scale ranging from 0 (never) to 4 (very often). The PSS-10 evaluated how unpredictable, uncontrollable, and overwhelming an individual considers their life to be over the past month. It was translated into Chinese and tested through internal consistency, the construct validity presenting good psychometric qualities.

### Salivary cortisol

Saliva samples were collected eight times per day for three consecutive weekdays, with strict reference to the time of morning awakening: 0, 15, 30, and 60 min after awakening, followed by four more samples at 3-h intervals throughout the day. Participants were given labeled 1.5-ml sterile Eppendorf tubes and adjustable alarm clocks to collect saliva samples. They were told not to eat, drink, chew gum, smoke, or brush their teeth 30 min before sampling and were instructed to place the cotton swabs under their tongues for at least 30 seconds and, when the swabs were saturated, to put them back into the Eppendorf tubes. Samples were stored in the participant’s home refrigerator until all saliva samples were collected, which were then shipped to our laboratory and stored at -80°C until assayed. Participants recorded the date, the precise wake-up time, and the time each sample was taken in a daily log. To ensure compliance with saliva collection procedures, we adjust the alarm clock to beep at designated times for saliva collection. In addition, notification information was sent to participants *via* mobile phone text messages the night before each sample collection. This non-invasive technique was used for home or work collection to minimize disruption to everyday daily life.

The free cortisol concentration in saliva was assayed in duplicate using a ^125^I spectral radioimmunoassay kit (Beijing North Institute of Biotechnology, Beijing, China), according to the manufacturer’s specifications. Assay sensitivity was estimated at 0.1 nmol/l. The intra- and inter-assay coefficients of variation were less than 6 and 10%, respectively. Unconverted cortisol values were used to calculate four cortisol measurements per day: the areas under the awakening response relative to dynamic increase (AUCi) and ground (AUCg) of cortisol awakening response (CAR), the diurnal cortisol slope (DCS) and diurnal cortisol AUCg. CAR AUCi indicated changes (positive or negative) in cortisol concentration, thus signifying HPA axis reactivity and response to arousal stress. However, the CAR AUCg value reflected the total cortisol secretion within 1h after awakening. DCS represented the change in cortisol secretion across the day, estimated by fitting a line that best matched all cortisol values. Finally, the diurnal AUCg showed the total salivary cortisol secretion and total HPA axis activity during the day. The three-day average of each cortisol measure was used in the analyses.

### Heart rate variability

By monitoring HRV, we could quantify the activity of the autonomic nervous system (ANS) and its sympathetic and parasympathetic modulation due to workload. The HRV was recorded using wireless Holter (BMS Century 3,000; Biomedical Systems, St. Louis, MO, United States) monitoring to obtain a 24-h electrocardiogram (ECG). During the recording, the participants followed their daily life and completed a time-activity diary. To avoid the influence of the circadian cycle, we scheduled all measurements to start at 7–8 a.m. and end at 24 h later. An ECG analysis software (CardioScan 12 Satellite; DM Software Inc., Beijing, China) was used to determine the frequency and time domain parameters of HRV. Arrhythmias and noise were automatically identified and filtered by the same software prior to the analysis of HRV parameters and then validated or modified, if necessary, by a trained cardiologist. Frequency-domain measures included: power in very low frequency (VLF; 0.01–0.04 Hz), in low frequency (LF; 0.04–0.15 Hz), in high frequency (HF; 0.15–0.40 Hz), and the LF/HF ratio. Time-domain parameters included: the standard deviation of normal-to-normal R-R intervals (SDNN), root mean square of the successive differences of R-R intervals (rMSSD), the proportion of the number of pairs of successive R-R intervals that differ by more than 50 ms divided by the total number of R-R intervals (pNN50) and standard deviation of the average R-R intervals calculated over 5 min (SDANN).

### Statistical analysis

SPSS 17.0 (SPSS Inc., Chicago, IL, United States) was used for all statistical analyses. Kolmogorov-Smirnov test, normal curve histogram, and P-P plots were used to test the normality of all continuous variables. Continuous variables were expressed as median and range or mean ± standard deviation (SD), depending on the (non-) normal distribution of the measured variables. Discontinuous variables were described as numbers (percentages). The independent-sample t-test was used to compare normal distribution continuous variables, and the Kruskal-Wallis test was used to compare non-normal variables. The IIEF-5 scores were adjusted for age because the ED is known to be age dependent. The chi-square test or fisher’s exact test was used for categorical variables.

Next, hierarchical linear regression analyses were conducted to examine whether the cortisol and HRV data were associated with perceived stress. A collinearity analysis was performed to check the prerequisites for regression analysis and showed no signs of collinearity (tolerance factor >0.1 and variance inflation factor<10). Due to concerns regarding multicollinearity, singular models tested the association between each stress system measure (HRV: VLF, HF, LF, LF/HR ratio, SDNN, SDANN, rMSSD, pNN50; Cortisol: CAR AUCi, CAR AUCg, diurnal AUCg, DCS) and perceived stress. Inter-relation models included the main effects and two-way interaction of HRV and cortisol measures. Simple slope analyses were used to interpret significant interactions. Tests were two-tailed, and *p*<0.05 was considered statistically significant.

## Results

### Demographic, symptomatic, and psychological characteristics

We recruited 92 patients with pEDs and 88 healthy men. Due to the withdrawal of consent, incomplete information, and other reasons, some participants discontinued the study, and finally, 75 pED patients and 75 healthy men completed our study. The ages of all subjects ranged from 20 to 49 (31.9 ± 7.1) years, with a median age of 30.5 years. The two groups were age-matched (*p*=0.418) and had a similar body mass index (BMI) (*p*=0.380). pEDs were all newly developed, and the disease course lasted from three to nine months, with a median of about five months. The distribution of occupational status (*p*<0.001) and marital status (*p*=0.007) were significantly different between the two groups. Compared with the control group, there were very few student patients and no single patients. Nevertheless, the distribution of educational attainment was roughly the same in both groups (*p*=0.129). Last but most important, the IIEF-5 and PSS-10 scores of pED patients were significantly different from those of healthy controls (*p*<0.001 for both). Although PSS-10 and IIEF-5 scores were negatively correlated in pED patients, there was no statistical significance between them (*r*=−0.049, *p*=0.677). The detailed characteristics of each group are presented in **Table 1**.

**Table 1:** Descriptive statistics: demographics, symptomatic and psychological scores, cortisol and HRV measures.

Characteristics and parameters	pED	Control	*p* value*
Demographics			
Sample size, n	75	75	N/A
Age (years), mean ± SD	31.44 ± 7.12	32.39 ± 7.15	0.418
BMI (kg/m^2^), mean ± SD	23.53 ± 1.80	23.83 ± 2.25	0.380
Employment, n(%)			<0.001
Student	3(4.0)	28(37.3)	
Employed	34(45.3)	21(28.0)	
Unemployed or retired	38(50.7)	26(34.7)	
Marital status, n(%)			0.007
Never married/single	0(0)	7(9.3)	
Married/cohabiting	73(97.3)	62(82.7)	
Divorced or separated	2(2.7)	6(8.0)	
Educational level, n(%)			0.129
Less than high school	13(17.3)	14(18.7)	
High school graduate/vocational school	14(18.7)	24(32.0)	
College graduate or higher education	48(64.0)	37(49.3)	
Symptomatic and psychological scores			
IIEF-5, mean ± SD	8.40 ± 2.34	23.12 ± 0.97	<0.001
PSS-10, mean ± SD	17.44 ± 4.75	13.97 ± 6.08	<0.001
Cortisol measures			
CAR AUCi (μg·min/dl), median (range)	85.20 (-123.98-486.83)	134.33(-550.80-1107.68)	0.744
CAR AUCg (μg·min/dl), median (range)	351.15 (93.00-577.20)	368.25 (93.00-1109.03)	0.972
Diurnal AUCg (μg·min/dl), median (range)	2211.45 (805.58-4834.13)	2111.63 (553.80-10345.88)	0.970
DCS (μg/dl/h), median (range)	-0.15(-0.48-0.11)	-0.05 (-1.05-0.18)	0.784
Frequency-domain HRV			
VLF (ms^2^), median (range)	2842.00 (146.00-6623.00)	2376.00 (240.00-5428.00)	0.219
LF (ms^2^), median (range)	670.00 (121.00-6289.00)	1893.00 (150.00-4876.00)	<0.001
HF (ms^2^), median (range)	622.00 (127.00-9624.00)	3845.00 (1179.00-7496.00)	<0.001
LF/HF, median (range)	0.66 (0.07-14.05)	0.60 (0.03-3.47)	0.014
Time-domain HRV			
SDNN (ms), median (range)	146.47 (29.36-327.23)	136.40 (30.62-274.38)	0.660
SDANN (ms), median (range)	118.96 (20.22-349.58)	113.52 (20.55-272.82)	0.866
rMSSD (ms), median (range)	37.25 (20.79-120.39)	58.68 (16.35-108.04)	0.065
pNN50 (ms), median (range)	16.50 (5.74-39.22)	18.50 (6.98-39.22)	<0.001

SD, standard deviation; BMI, body mass index; IIEF-5, International Index of Erectile Function-5; PSS-10, Perceived Stress Scale-10; CAR, cortisol awakening response; AUCi, area under the curve with respect to increase; AUCg, area under the curve with respect to ground; DCS, diurnal cortisol slope; HRV, heart rate variability; VLF, very low frequency; LF, low frequency; HF, high frequency; SDNN, standard deviation of all R-R intervals; SDANN, standard deviation of the average R-R intervals calculated over 5 min; rMSSD, root mean square of successive differences; pNN50, percent of R-R intervals differing more than 50 ms from each other; pED, psychogenic erectile dysfunction.

*p value from an independent-sample t-test for age, BMI, symptom, and psychological scores, Kruskal-Wallis test for salivary cortisol and HRV values, and the chi-square test or fisher’s exact test for discontinuous variables. N/A, not applicable.

### Measurement of cortisol and HRV parameters

**Table 1** also showed the differences in cortisol and HRV parameters between groups. In the frequency domain analysis, the LF and HF power of the pED group were significantly lower than those of the control group (both *p*<0.001), but the pED patients had a significantly higher LF/HF ratio than the controls (*p*=0.014). In the time domain analysis, only pNN50 was significantly higher in the healthy control group than in the pED group (*p*<0.001). However, no statistically significant differences in changes between groups were seen in any cortisol variables during the study (all *p*>0.05).

### Singular versus inter-relation models

A two-way interaction term tested whether each HRV parameter moderated the relation between cortisol measures and perceived stress. In pED patients, CAR AUCi, CAR AUCg, and diurnal cortisol AUCg demonstrated significant associations with perceived stress in the models. Inter-relation models with the interactions of HF*CAR AUCi and rMSSD*CAR AUCi accounted for significantly greater variance in perceived stress than the singular HRV or cortisol models. Other parameters had no significant interactions. The inter-relation of HF*CAR AUCi and rMSSD*CAR AUCi accounted for an additional 6.1% and 5.9% of the variance in perceived stress. Results are given in **Table 2**. However, in the control group, only pNN50 was associated with perceived stress in the singular HRV model. No significant interactions were found in all HRV and cortisol parameters (**Table 3**).

**Table 2:** Singular versus inter-relation model comparisons in pED patients.

HRV	Cortisol	Singular models HRV			Singular models cortisol			Inter-relation models					Model comparison	
		β_hrv_	F	R^2^	β_cort_	F	R^2^	β_hrv_	β_cort_	β_int_	F	R^2^	ΔF	ΔR^2^
VLF		-0.080	0.465	-0.007	–	–	–	–	–	–	–	–	–	–
	CAR AUCi	–	–	–	0.183	2.538	0.020	-0.033	0.189	0.079	1.081	0.003	0.334	0.005
	CAR AUCg	–	–	–	0.263*	5.412	0.056	-0.093	0.275*	-0.054	1.920	0.036	0.142	0.002
	Diurnal AUCg	–	–	–	0.217	3.617	0.034	-0.003	0.217	0.048	1.238	0.010	0.122	0.002
	DCS	–	–	–	-0.075	0.413	-0.008	-0.094	-0.095	0.024	0.348	-0.027	0.038	0.001
LF		0.153	1.740	0.010	–	–	–	–	–	–	–	–	–	–
	CAR AUCi	–	–	–	–	–	–	0.046	0.203	0.127	1.403	0.016	0.877	0.012
	CAR AUCg	–	–	–	–	–	–	0.067	0.241	0.005	1.872	0.034	0.001	0.000
	Diurnal AUCg	–	–	–	–	–	–	0.196	0.291	0.108	2.005	0.039	0.430	0.006
	DCS	–	–	–	–	–	–	0.142	-0.058	-0.008	0.643	-0.015	0.004	0.000
HF		0.069	0.353	-0.009	–	–	–	–	–	–	–	–	–	–
	CAR AUCi	–	–	–	–	–	–	-0.108	0.384*	0.348*	2.545	0.059	4.798	0.061
	CAR AUCg	–	–	–	–	–	–	-0.062	0.312*	0.124	1.947	0.037	0.489	0.006
	Diurnal AUCg	–	–	–	–	–	–	0.154	0.399*	0.217	1.904	0.035	1.363	0.018
	DCS	–	–	–	–	–	–	0.065	-0.054	0.025	0.216	-0.033	0.033	0.000
LF/HF		-0.052	0.199	-0.011	–	–	–	–	–	–	–	–	–	–
	CAR AUCi	–	–	–	–	–	–	-0.165	0.252	-0.198	1.464	0.018	1.651	0.022
	CAR AUCg	–	–	–	–	–	–	-0.018	0.261*	-0.002	1.762	0.030	0.000	0.000
	Diurnal AUCg	–	–	–	–	–	–	-0.002	0.213	0.015	1.179	0.007	0.005	0.000
	DCS	–	–	–	–	–	–	0.115	-0.064	-0.234	0.934	-0.003	2.282	0.031
SDNN		0.081	0.481	-0.007	–	–	–	–	–	–	–	–	–	–
	CAR AUCi	–	–	–	–	–	–	0.031	0.171	-0.028	0.878	-0.005	0.053	0.001
	CAR AUCg	–	–	–	–	–	–	-0.015	0.268*	-0.043	1.814	0.032	0.143	0.002
	Diurnal AUCg	–	–	–	–	–	–	0.087	0.231	0.055	1.390	0.016	0.195	0.003
	DCS	–	–	–	–	–	–	0.041	-0.059	-0.077	0.365	-0.026	0.369	0.005
SDANN		0.074	0.406	-0.008	–	–	–	–	–	–	–	–	–	–
	CAR AUCi	–	–	–	–	–	–	0.016	0.167	-0.099	1.087	0.004	0.688	0.009
	CAR AUCg	–	–	–	–	–	–	-0.009	0.263*	-0.061	1.866	0.034	0.263	0.003
	Diurnal AUCg	–	–	–	–	–	–	0.069	0.223	0.037	1.302	0.012	0.090	0.001
	DCS	–	–	–	–	–	–	0.033	-0.063	-0.069	0.321	-0.028	0.284	0.004
rMSSD		0.009	0.006	-0.014	–	–	–	–	–	–	–	–	–	–
	CAR AUCi	–	–	–	–	–	–	-0.059	0.274*	0.266*	2.424	0.055	4.640	0.059
	CAR AUCg	–	–	–	–	–	–	-0.007	0.254*	0.041	1.794	0.031	0.109	0.001
	Diurnal AUCg	–	–	–	–	–	–	0.137	0.301*	0.188	1.987	0.038	2.063	0.027
	DCS	–	–	–	–	–	–	-0.005	-0.076	-0.009	0.136	-0.036	0.006	0.000
pNN50		0.086	0.548	-0.006	–	–	–	–	–	–	–	–	–	–
	CAR AUCi	–	–	–	–	–	–	0.051	0.202	0.099	1.116	0.005	0.664	0.009
	CAR AUCg	–	–	–	–	–	–	0.065	0.239*	-0.063	1.936	0.037	0.277	0.004
	Diurnal AUCg	–	–	–	–	–	–	0.157	0.280*	0.096	1.769	0.030	0.526	0.007
	DCS	–	–	–	–	–	–	0.073	-0.090	-0.037	0.339	-0.028	0.071	0.001

HRV, heart rate variability; VLF, very low frequency; LF, low frequency; HF, high frequency; SDNN, standard deviation of all R-R intervals; SDANN, standard deviation of the average R-R intervals calculated over 5 min; rMSSD, root mean square of successive differences; pNN50, percent of R-R intervals differing more than 50 ms from each other; CAR, cortisol awakening response; AUCi, area under the curve with respect to increase; AUCg, area under the curve with respect to ground; DCS, diurnal cortisol slope; β_hrv_, standardized beta coefficient for HRV measure; β_cort_, standardized beta coefficient for cortisol measure; β_int_, standardized beta coefficient for the interaction term. Bold values indicate significance: *p < 0.05.

**Table 3:** Singular versus inter-relation model comparisons in healthy men.

HRV	Cortisol	Singular models HRV			Singular models cortisol			Inter-relation models					Model comparison	
		β_hrv_	F	R^2^	β_cort_	F	R^2^	β_hrv_	β_cort_	β_int_	F	R^2^	ΔF	ΔR^2^
VLF		0.129	1.231	0.003	–	–	–	–	–	–	–	–	–	–
	CAR AUCi	–	–	–	0.034	0.082	-0.013	0.128	0.024	0.014	0.418	-0.024	0.014	0.000
	CAR AUCg	–	–	–	0.091	0.610	-0.005	0.129	0.093	0.059	0.719	-0.012	0.254	0.003
	Diurnal AUCg	–	–	–	0.131	1.279	0.004	0.131	0.148	0.066	1.029	0.001	0.315	0.004
	DCS	–	–	–	-0.095	0.658	-0.005	0.130	-0.103	-0.076	0.825	-0.007	0.415	0.006
LF		-0.136	1.372	0.005	–	–	–	–	–	–	–	–	–	–
	CAR AUCi	–	–	–	–	–	–	-0.141	0.054	-0.010	0.509	-0.020	0.006	0.000
	CAR AUCg	–	–	–	–	–	–	-0.132	0.077	0.035	0.647	-0.015	0.088	0.001
	Diurnal AUCg	–	–	–	–	–	–	-0.115	0.117	-0.026	0.750	-0.010	0.048	0.001
	DCS	–	–	–	–	–	–	-0.119	-0.052	-0.038	0.584	-0.017	0.085	0.001
HF		0.042	0.131	-0.012	–	–	–	–	–	–	–	–	–	–
	CAR AUCi	–	–	–	–	–	–	0.037	0.057	-0.041	0.110	-0.037	0.088	0.001
	CAR AUCg	–	–	–	–	–	–	0.044	0.072	0.082	0.386	-0.026	0.459	0.006
	Diurnal AUCg	–	–	–	–	–	–	0.009	0.065	0.237	1.773	0.030	3.827	0.050
	DCS	–	–	–	–	–	–	0.009	-0.074	-0.038	0.246	-0.032	0.078	0.001
LF/HF		-0.108	0.855	-0.002	–	–	–	–	–	–	–	–	–	–
	CAR AUCi	–	–	–	–	–	–	-0.108	0.050	0.022	0.315	-0.029	0.022	0.000
	CAR AUCg	–	–	–	–	–	–	-0.098	0.030	-0.078	0.518	-0.020	0.272	0.004
	Diurnal AUCg	–	–	–	–	–	–	-0.069	0.053	-0.147	1.047	0.002	1.066	0.014
	DCS	–	–	–	–	–	–	-0.101	-0.012	0.114	0.636	-0.015	0.654	0.009
SDNN		0.111	0.910	-0.001	–	–	–	–	–	–	–	–	–	–
	CAR AUCi	–	–	–	–	–	–	0.093	0.036	-0.069	0.407	-0.025	0.310	0.004
	CAR AUCg	–	–	–	–	–	–	0.124	0.068	0.091	0.698	-0.012	0.555	0.008
	Diurnal AUCg	–	–	–	–	–	–	0.141	0.154	0.131	1.356	0.014	1.283	0.017
	DCS	–	–	–	–	–	–	0.092	-0.072	-0.136	1.007	0.000	1.201	0.016
SDANN		0.097	0.695	-0.004	–	–	–	–	–	–	–	–	–	–
	CAR AUCi	–	–	–	–	–	–	0.077	0.043	-0.092	0.421	-0.024	0.555	0.008
	CAR AUCg	–	–	–	–	–	–	0.109	0.079	0.068	0.547	-0.019	0.309	0.004
	Diurnal AUCg	–	–	–	–	–	–	0.138	0.155	0.114	1.139	0.006	0.951	0.013
	DCS	–	–	–	–	–	–	0.078	-0.068	-0.158	1.090	0.004	1.652	0.022
rMSSD		0.156	1.813	0.011	–	–	–	–	–	–	–	–	–	–
	CAR AUCi	–	–	–	–	–	–	0.160	0.013	0.037	0.631	-0.015	0.089	0.001
	CAR AUCg	–	–	–	–	–	–	0.148	0.061	0.045	0.796	-0.008	0.118	0.002
	Diurnal AUCg	–	–	–	–	–	–	0.164	0.144	0.069	1.290	0.012	0.341	0.005
	DCS	–	–	–	–	–	–	0.175	-0.119	0.114	1.066	0.003	0.845	0.011
pNN50		0.244*	4.626	0.047	–	–	–	–	–	–	–	–	–	–
	CAR AUCi	–	–	–	–	–	–	0.247*	0.016	0.016	1.518	0.021	0.014	0.000
	CAR AUCg	–	–	–	–	–	–	0.249*	0.000	0.147	2.085	0.042	1.234	0.016
	Diurnal AUCg	–	–	–	–	–	–	0.235*	0.070	0.154	2.565	0.060	1.469	0.019
	DCS	–	–	–	–	–	–	0.238*	-0.079	-0.006	1.681	0.027	0.002	0.000

HRV, heart rate variability; VLF, very low frequency; LF, low frequency; HF, high frequency; SDNN, standard deviation of all R-R intervals; SDANN, standard deviation of the average R-R intervals calculated over 5 min; rMSSD, root mean square of successive differences; pNN50, percent of R-R intervals differing more than 50 ms from each other; CAR, cortisol awakening response; AUCi, area under the curve with respect to increase; AUCg, area under the curve with respect to ground; DCS, diurnal cortisol slope; β_hrv_, standardized beta coefficient for HRV measure; β_cort_, standardized beta coefficient for cortisol measure; β_int_, standardized beta coefficient for the interaction term. Bold values indicate significance: *p < 0.05.

### Simple slope test

**Figure 1:**
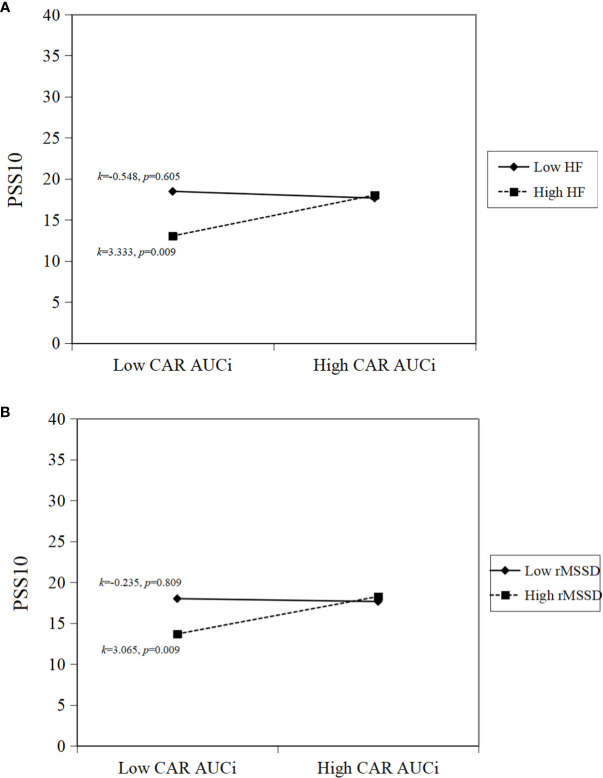
Simple slope interpretations of significant inter-relations. Higher sympathovagal modulation and higher cortisol awakening response are associated with greater perceived stress. In the panels, **(A)** higher HF and higher CAR AUCi; **(B)** higher rMSSD and higher CAR AUCi, are associated with higher perceived stress.

A simple slope analysis was performed to identify patterns of the inter-relations between ANS and HPA axis activity and to show how HF and rMSSD modulated the association between CAR AUCi and perceived stress in pED patients. Specifically, higher HF combined with higher CAR AUCi was associated with higher perceived stress (*k*=3.333, *p*=0.009; **Figure 1A**). Similarly, higher rMSSD combined with greater CAR AUCi was associated with higher perceived stress (*k*=3.065, *p*=0.009; **Figure 1B**).

## Discussion

Psychophysiological measures are reliable indicators of stress. PSS-10 is a widely used psychological tool to assess non-specific perceived chronic stress and to measure the extent to which a person’s life situation is evaluated as stressful (21). As expected, pED patients in this study had significantly higher PSS-10 scores than age-matched asymptomatic men, suggesting that pED patients did experience more significant perceived stress; however, their perceived stress levels were not correlated to pED severity. pED patients with the most severe symptoms did not necessarily show the highest perceived stress levels, and other psychosocial factors may also be responsible for pED.

Recent studies have confirmed a close correspondence between perceived stress levels and physiological or hormonal stress (HRV and cortisol) parameters (22, 23). Our results showed that compared with the control group, in addition to the significant LF/HF ratio increase, the LF, HF, and pNN50 were significantly decreased in pED patients. However, no changes in cortisol parameters were observed. Mathematically, the significant increase in LF/HF ratio may be due to the greater power decline of HF than LF. Chen et al. (17) reported strikingly congruent results to our findings. They found that patients with non-organic ED had significantly lower HF and significantly higher LF/HF than the healthy group. However, in patients with erectile problems, only a statistically significant increase in LF/HF ratio was found (16). LF reflects parasympathetic and sympathetic nervous system activity, and HF mainly reflects vagal activity. LF/HF estimates the relative balance between sympathetic and parasympathetic response; rMSSD and pNN50 mainly reflect the tension of the parasympathetic nerve (24). In the present study, the decrease of LH, HF, and pNN50 and the increase of LF/HF ratio reconfirmed the theory that there might be an ANS imbalance in pED patients, which was mainly manifested as impaired parasympathetic tone, and ultimately, the imbalance was still sympathetic dominance (16, 17, 24).

Salivary cortisol, an indicator of the hormonal stress system, is usually considered a reliable and non-invasive marker for assessing HPA axis activity (25). Most studies have described increased activation of the HPA axis in response to various stressors (26–28). However, our results showed no significant differences in the CAR and diurnal cortisol profiles between pED patients and healthy individuals. Three possible reasons may explain the lack of change in pED patients’ HPA axis activity. First, we speculated that acute or physical stress was more likely to stimulate the hyperactivity of the HPA axis. However, chronic mental stress associated with pED could keep the basal activity of the HPA axis intact (25). Second, since the pED patients we recruited were new cases with a relatively short course of the disease, the accumulation of stressors was insufficient to activate the HPA axis. Third, a smaller sample size may not capture subtle changes in HPA axis activity during statistical analysis.

The above results emphatically analyze the changes in psychophysiological parameters related to stress in pED patients compared with healthy men. However, the relationship between perceived stress and the stress response system is still unclear. In the current study, the results of regression analyses partially supported the hypothesis that the inter-relation between measures of ANS (HF, rMSSD) and the HPA axis (CAR AUCi) in pED patients was better associated with perceived stress than the singular effect of either ANS or HPA axis activity. Meanwhile, significant interactions between rMSSD or HF and CAR AUCi accounted for a relatively high 5.9% or 6.1% variance in perceived stress. In some child and adolescent psychology studies, it is also suggested that there is an interrelation between autonomic and HPA axis activity. Rotenberg et al. (18) found that the interaction of cardio-autonomic control and the HPA axis accounted for 2–4% of the variance in perceived stress. El-Sheikh et al. (29) reported that the interaction of HF and cortisol accounted for 2-14% of the variance in anxiety and depressive symptoms. These results, including ours, support emerging theories in stress physiology, which emphasize the importance of considering the interrelation between the physiological components of the stress response system (18, 30). Interestingly, no significant interaction between HRV and cortisol measures related to perceived stress was found in healthy individuals. It is inferred indirectly that the distribution of PSS-10 scores and physiological or hormonal stress parameters in healthy men is more concentrated and tight, and the distribution range is narrower.

Because HF and rMSSD reflect parasympathetic or vagus nerve activity and CAR AUCi represents HPA axis response and arousal stress response, simple slope analyses of significant interactions in the present study indicated that pED patients with higher parasympathetic activity and cortisol awakening response had greater perceived stress. On the contrary, it could be understood that, with the decrease in parasympathetic activity, the effect of HPA axis activity on perceived stress in pED patients was gradually weakened. This finding is another explanation for changes in HRV and cortisol parameters in pED patients compared to normal men. Namely, the impaired parasympathetic tone in pED patients resulted in the attenuated influence of the HPA axis on perceived stress. Therefore, sympathovagal modulation played a major role in regulating perceived stress. Park et al. (31) reported that forest environments could lower cortisol concentrations, lower pulse rate, lower blood pressure, increase parasympathetic nerve activity, and lower sympathetic nerve activity compared with city settings, and finally aid in effectively relaxing the human body. Vieira et al. (32) found that auriculotherapy therapy could relieve university students’ anxiety before exams by activating the parasympathetic nervous system and reducing salivary cortisol levels. Moreover, yoga stretching enhanced parasympathetic activity and decreased salivary cortisol levels, which could compensate for the lack of exercise and increase life expectancy in the general population (33). These researches suggest that if we increase parasympathetic activity in pED patients while maintaining normal levels of stress hormones, it may be possible to reduce perceived stress and even promote penile erection.

Our study has the following limitations. First, we could not verify and compare our findings due to the lack of other studies on the relationship between HPA axis activity and perceived stress in patients with ED (34). Comparisons with other stress-related studies in specific populations are difficult because we used hierarchical multiple linear regression analysis, interaction effect analysis, and simple slope analysis, which may have increased our chances of finding meaningful associations between perceived stress and stress response systems. Second, more measures are needed to evaluate the stress and the activity of the ANS and HPA axis. In addition to perceived stress, chronic stress and stressful life events are also associated with adverse outcomes (35). Future research should consider measuring stress from a multidimensional perspective (e.g., longer duration, early life adversity) (18). Furthermore, we used only HRV and salivary cortisol to assess the ANS and HPA axis activity. Future studies should consider other autonomic measures (e.g., salivary alpha-amylase, pre-ejection period) and other stress hormones (e.g., cortisone, dehydroepiandrosterone) to more comprehensively characterize the ANS and HPA axis (15, 18). In summary, future research would benefit from extending the current findings by examining how the inter-relation between the ANS and the HPA axis is associated with other stress components, a larger sample size over a more extended period, and using additional measures of autonomic and HPA activity. Third, because the analyses were cross-sectional, our results cannot determine any causal direction for the associations found. Future longitudinal studies for pED patients are necessary to investigate further the interrelation between autonomic and HPA axis functioning and its relationship with stress (25).

In conclusion, we first studied the changes in perceived stress and two neural stress systems in pED patients. Then we provided evidence for the importance of considering the inter-relation between the ANS and the HPA axis when investigating the relationship between stress and the stress response system. Our descriptive statistics found that the PSS-10 score of pED patients was significantly higher than that of healthy controls, and there was no correlation with the IIEF-5 score. In terms of the stress response system, our results demonstrated that compared with healthy individuals, the HRV parameters of pED patients decreased. However, the cortisol parameters did not change, suggesting that the stress response of pED patients was characterized by ANS imbalance rather than HPA dysregulation. The ANS imbalance was mainly caused by significantly impaired parasympathetic vagal tone (decreased HF and pNN50 index), which eventually led to the dominance of sympathetic tone (increased LF/HF ratio). Moreover, regression analyses showed that the interrelation between ANS and the HPA axis was more related to perceived stress than the singular correlation of either the ANS or HPA axis. pED patients with higher parasympathetic activity and a higher cortisol awakening response had greater perceived stress. These results suggest that the interaction between the ANS and HPA axis is uniquely related to perceived stress. Identifying different patterns of stress responses and linking these patterns to adverse health outcomes is vital to advance the current understanding of how stress affects the physical and mental health of pED patients. Further large-scale, longitudinal, multi-center investigations and animal experiments might confirm and generalize our findings.

## Data availability statement

The original contributions presented in the study are included in the article/**Supplementary Material**. Further inquiries can be directed to the corresponding author.

## Ethics statement

The studies involving human participants were reviewed and approved by the Institutional Review Board of Tongji Hospital. The patients/participants provided their written informed consent to participate in this study.

## Author contributions

SW, JL, LJ, and JB contributed to the conception and design of the study. ML, BH, SY, YC, and XD collected clinical data and specimens. KR, ML, JY, and XL analyzed the data. JX wrote the first draft of the manuscript. TW, YC, KR, and LG wrote sections of the manuscript. All authors contributed to the article and approved the submitted version.
